# Exercise in multiple sclerosis -- an integral component of disease management

**DOI:** 10.1007/s13167-011-0136-4

**Published:** 2011-12-24

**Authors:** Andrea Döring, Caspar F Pfueller, Friedemann Paul, Jan Dörr

**Affiliations:** 1 National Representative of EPMA in Germany; 2 NeuroCure Clinical Research Center and Clinical and Experimental Research Center for Multiple Sclerosis, Charité - Universitätsmedizin Berlin, Charitéplatz 1, 10117 Berlin, Germany

**Keywords:** Multiple sclerosis, Physical therapy, Exercise, Prevention of sequelae, Personalized treatment

## Abstract

Multiple sclerosis (MS) is the most common chronic inflammatory disorder of the central nervous system (CNS) in young adults. The disease causes a wide range of symptoms depending on the localization and characteristics of the CNS pathology. In addition to drug-based immunomodulatory treatment, both drug-based and non-drug approaches are established as complementary strategies to alleviate existing symptoms and to prevent secondary diseases. In particular, physical therapy like exercise and physiotherapy can be customized to the individual patient's needs and has the potential to improve the individual outcome. However, high quality systematic data on physical therapy in MS are rare. This article summarizes the current knowledge on the influence of physical activity and exercise on disease-related symptoms and physical restrictions in MS patients. Other treatment strategies such as drug treatments or cognitive training were deliberately excluded for the purposes of this article.

## Background of MS

MS is a chronic inflammatory disease of the CNS, which causes multifocal demyelination along with astrocytic gliosis and variable axon loss in the brain and spine. MS is one of the most common causes of non-traumatic disability in young adults and approximately 1-2.5 million people around the world are estimated to be affected, depending on the publication [1,2]. Women are more likely to develop the disease than men (female:male ratio approximately 2-3:1). MS usually manifests between the age of 20 to 40 years, rarely much earlier during childhood, or in old age. The disease course is usually relapsing-remitting with progression into a secondary progressive form after a varying period of time or primary progressive right from the start. The precise etiology of MS still remains unclear. A combination of environmental and genetic factors which lead to autoimmune reactions against CNS-structures which in turn result in CNS tissue damage and neurological impairment is assumed to be the most likely pathomechanism [2,3].

Depending on the localization and characteristics of the morphological changes in both white and gray brain matter, different symptoms and signs may occur, such as visual impairment, dysarthria and dysphagia, spasticity, paresis, coordination and balance impairment, ataxia, pain, sensory impairment, bladder, bowel and sexual dysfunction [3-7]. Fatigue, emotional and cognitive changes are also frequently present in MS [8-13]. These symptoms, often in combination with a lack of confidence in one's own capabilities and abilities to manage the symptoms, lead to impaired functional capacity and subsequently reduced physical and sporting activity as well as reduced quality of life [14-18]. As in other conditions with reduced mobility, in MS the lack of physical activity can lead to secondary sequelae such as obesity, osteoporosis, and/or cardiovascular damage which in turn pose a serious threat to patients as they increase the risk of further complications like thrombosis, pulmonary embolisms, upper respiratory or urinary tract infections, or prominent decubital ulcers [15,16,19].

**Figure 1:**
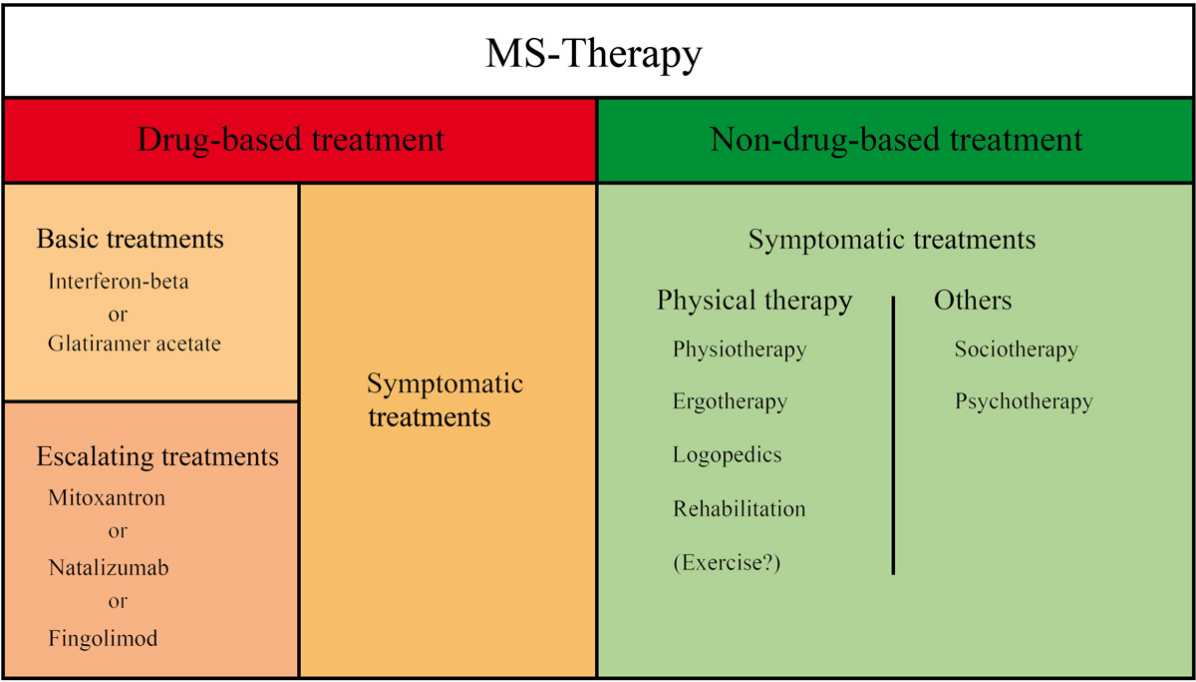
**Drug-based and non-drug-based symptomatic treatment approaches for MS complement each other in almost every stage of disease**. Drug-based strategies encompass basic treatments (interferon-β or glatriameracetete) and -- if these drugs are not sufficiently effective -- escalation therapy with immunosuppressive substances (mitoxantrone), monoclonal antibodies (natalizumab) or sphingosinphosphat receptor modulator fingolimod. Non-drug strategies like physical therapy (physiotherapy, ergotherapy, logopedics, rehabilitaton) and occupational therapy (sociotherapy and psychotherapy) are used complementarily in all stages of the disease

According to the autoimmune etiopathology, immunomodulatory drugs such as interferon-β or glatiramer acetate are the treatment of choice. If these drugs are not sufficiently effective, escalation therapy with immunosuppressive substances (mitoxantrone), monoclonal antibodies (natalizu-mab) or the recently approved sphingosinphosphat receptor modulator fingolimod may be required (Figure 1) [20-22].

## Definitions

For the purpose of this article the terms movement, physical activity, exercise, physical function, physical therapy, physiotherapy and sport will be used according to the following definitions (Tables 1 and 2): In terms of the motor system, the term "movement" includes an actively or passively induced change in the position of the body. Regular exercise and physical activity are decisive factors in a person's quality of life by sustainably improving health and wellbeing and preventing diseases at all stages of life. As opposed to sport, in which the focus is on physical achievement, competition and fun, physical activity encompasses any type of physical movements, which consume energy, regardless of the underlying motivation. The term "health-enhancing physical activity" includes both leisure-time activities (e.g. sport) and everyday activities (e.g. climbing stairs). The intensity of the activity is categorized according to the metabolic equivalent (MET; 1 MET corresponds to the oxygen uptake of an adult whilst sitting = 3.5 ml (men) and 3.2 ml (women) O_2_/kg/min) into light (<3 MET), moderate (3-6 MET) and vigorous (>6 MET). In contrast to general physical activity, exercise encompasses the planned performance of systematically repeated movements to accomplish skills, maintain and strengthen physical condition, and improve performance. Athletics, more specifically, aims to improve general flexibility and includes endurance training to maintain performance over longer periods of time at a high level and strength training to increase muscle strength. The terms endurance and aerobic training as well as resistance and strength training are often used synonymously. Physical function encompasses "*a series of increasingly integrated steps, with the highest level consisting of the most advanced activities of daily life (ADL), the fulfillment of societal roles and the pursuit of recreational activities*" [16]. The term "physiotherapy" includes manual skills, that are appropriately supplemented by remedies like water, heat, light, or electricity and aims to restore functionality and conscious perception of the human body. Active and/or passive training programs are part of physiotherapeutic methods. On the contrary "physical therapy" is rather used as an umbrella-term, comprising different kinds of physical activity like exercise, (functional) training, physiotherapy, and rehabilitation.

**Table 1:** Definitions of different types of physical movements

**Definitions**	
Movement	Active or passive change in the position of the body
Physical activity	Any type of physical movement that consumes the subject's energy
Physical function	A series of increasing steps, with the highest level consisting of the most advanced activities of daily life, the fulfillment of social roles and the pursuit of recreational activities
Exercise	Planned performance of systematically repeated movements to accomplish skills, maintain and strengthen physical condition, and improve performance
Sport	Exercise with a focus on physical achievement, competition, and fun

**Table 2:** Types of human movements, sorted according to intensity

Type of human movements
Movement → Physical activity → Physical function → Exercise → Sport

## Symptomatic treatment of MS -- aiming at a personalized modification of symptoms and outcome

Drug-based and non-drug-based symptomatic treatment approaches for MS complement each other. Drug-based approaches which are referred to in comprehensive reviews [21,22] are beyond the scope of this article. Apart from counseling and nursing care, non-drug strategies encompass physical therapy like physiotherapy, logopedics, occupational therapy including living and mobility aids, sociotherapy and psychotherapy (Figure 1). These measures can be applied multimodally, meaning that several approaches are combined in a patient's treatment strategy and should generally complement drug therapy [4,23,24]. Physical therapies are developed depending on the individual symptoms and positively affect several factors at the same time. Importantly, apart from reducing symptoms, enhancing mobility, improving quality of life and conferring as much independence as possible, for example by functional training of ADLs, such as washing, eating, drinking, dressing, and performing household chores, symptomatic therapies may prevent potentially life-threatening secondary diseases [15,25]. Physical therapies can be applied in almost every stage of disease -- from the first onset of symptoms to highly impaired patients and palliative conditions. In contrast to physiotherapy, exercise is not part of commonly used therapies offered to MS patients; however it might be a promising and cost-effective tool to improve various functions in patients with MS.

## Exercise in MS patients -- effects on clinical parameters (Table 3)

**Table 3:** Overview of selected studies on exercise in multiple sclerosis

Form of training	Author	Method	(Major) Endpoints	Sample size	EDSS	Main Results	Comments
Aerobic training	Newman [27]	Treadmill walking	Gait parameters, Fatigue	16	<7	Improvements in some gait parameters, fatigue unchanged	Repeated measures design and blinded assessments
	Pilutti [32]	Body-weight supported treadmill training (BWSTT)	Functional ability, quality of life, Fatigue	6	5,5-8	Improvements in some functional abilities and some parameters of quality of life, Fatigue non-significantly reduced	Patients with progressive MS of high disability
	Rampello [42]	Aerobic training program compared with neurological rehabilitation	Walking parameters, maximal exercise tolerance, quality of life, fatigue	19	< 6	Improvements of some walking parameters after aerobic training, Fatigue after aerobic training and neurological rehabilitation comparable	Only 11 patients completed
	Schulz [44]	Aerobic training	Immune-endocrine parameters, neurotrophic factors, quality of life, coordinative function	67	<5	Lactate levels lowered, quality of life increased and coordinative increased	
	Van den Berg [47]	Aerobic treadmill training	Walking parameters, fatigue	19	Walk 10 m in 60 s, using aid if necessary	Improvements of some walking parameters after aerobic treadmill training, fatigue not significantly reduced	Prospective, randomized controlled trial with blinded assessments, 16 patients completed
	Mostert [48]	Aerobic training	Aerobic fitness, fatigue, health perception, activity level	26	<6.5	Improvement of health perception, activity level	Randomized trial
Resistance Training	Dodd [28]	Progressive resistance strengthening	Physical, psychological, social factors	8	no information	Fatigue reduced significantly	Semi-structured interviews
	Dalgas [31]	Progressive resistance training	Muscle strength, functional capacity	38	3-5.5	Muscle strength and functional capacity increased	Randomized controlled trial including follow-up
	Harvey [39]	General physiotherapy exercises, strengthening training	Muscle strength, functional activities	19	Ambulant with or without the use of walking aids	Muscle strength and functional activities improved	
	White [30]	Progressive resistance training	Lower extremity strength, ambulatory function, fatigue, disability	8	1-5	Lower extremity strength increased	
	Taylor [40]	Progressive resistance training	Maximal muscle force, muscle endurance, functional activity, psychological function	9	able to walk at least 200 m without aid or rest	Improvements in muscle strength, muscle endurance, and functional activity	Pre/post single group research design
	Gutierrez [49]	Resistance training	Kinematic gait parameters, isometric strength, stepping, fatigue, disability	8	2.5-5.5	Increases in some kinematic gait parameters, fatigue decreased	
	Broekmans [101]	Resistance training	Muscle strength, functional mobility	36	2-6.5	Improvements in muscle strength and some functional parameters	Randomized controlled trial, long-term investigation (20 weeks)
Combined training	Romberg [34]	Strength, aerobic training	Walking speed, lower extremity strength, upper extremity endurance, dexterity, static balance	95	1-5.5	Walking speed improved	6-month exercise program, randomized study, 91 patients completed
	Cakit [35]	Cycling progressive resistance training, balance exercises	Walking parameters, balance, fatigue, fear of falling, depression, quality of life	45	of ≤ 6.0, ability to stand independently in upright position for >3 s	Improvements in walking parameters, fatigue, fear of falling, depression	Randomized (two exercise training and one control group), only 33 patients completed
	Smith [46]	Strengthening, stretches, fitness exercises	Function, fatigue, sensory symptoms	34	0-6	> 40% temporary increased sensory symptoms (number or intensity)	Single exercise session with follow up, all measures self-rated
	Surakka [50]	Aerobic and strength exercise	Motor fatigue	95	1-5.5	Motor fatigue reduced in women (not in men)	Randomized controlled trial
Others	Motl [17]	Wearing an accelerometer	Physical activity, quality of life, disability, fatigue, mood, pain, self-efficacy, social support	292	no information	Improvements in disability, fatigue, depression, pain, self-efficacy, social support	No specific training-protocol, completed self- report measures
	Rasova [43]	Neurophysiologically based physiotherapy, aerobic training, combined therapy	Impairment, disability, handicap, quality of life, fatigue, depression, respiratory function, physical fitness	112	0-6.5	Improvements in training groups with different impact on parameters, fatigue reduced	Randomized (three exercise training and one control group), only 95 patients completed
	Wiles [51]	Physiotherapy	Mobility, mood	42	4.0-5.5	Improvements in mobility, subjective wellbeing, and mood	Randomized controlled crossover trial

Impairment of MS patients like spasticity or paresis is primarily a consequence of disease progress (morphological changes), but it can be aggravated by reduced physical activity [14,26]. Exercise has been shown to improve various aspects of the physiological profile of MS patients; in particular, inactivity-related impairment can be alleviated by exercise [26]. However, recommendations on exercise for patients with MS have to face a number of limitations: Although there is a large number of studies on which recommendations have been based, many of these studies have limitations, including small sample sizes, lack of an appropriate control group, unblinded design, and failure to distinguish between different courses and stages of the disease. In fact, only occasionally a randomized controlled and blinded study design is applied. Training regimes are often not standardized, and the interventions are hardly sufficiently described. The comparability of studies is furthermore limited by variable treatment duration extending over a short period of weeks up to few months, different treatment frequency and different treatment intensity. Long term-effects of the respective interventions are rarely reported [14,27-31]. Furthermore, effects of exercise have been studied almost exclusively in MS patients with slight or moderate impairment (score on the expanded disability status scale (EDSS) less than 7) [14]. To our knowledge only one recently published study examined highly impaired MS patients with an EDSS of 5-8 [32].

In summary, despite the often insufficient methodological quality of the studies and the insufficiently described training regimes [14,29,33] most of these studies including exercise programs of resistance (e.g. progressive resistance exercise, walking mechanics), endurance (e.g. bicycle ergometry, arm or arm-leg ergometry, aquatic exercise, treadmill walking) as well as combined training provided evidence for a benefit of exercise in MS patients [14,15,28,29]. These training programs are referred to in more detail below. All training programs have been well tolerated by the patients. Nearly 100% of inpatient participants and 59-96% participants of home-based trials completed without occurrence of adverse events [34-38].

### Endurance training

Moderate endurance training resulted in improved muscle strength of both lower and upper extremities and some functional measures like walking speed, fatigue, and quality of life [14,15,17,28,29,31,34]. Some authors reported beneficial effects in chair transfer [14,39], gait, stair climbing, and timed up and go test (standing up from a chair, walking 3 m, turning around and seat again) [14,35,40]. But, as described above, varying and contradictory results were found. For example, some authors reported marked improvements in aerobic capacity, measured by maximal oxygen uptake (VO_2_-max), [14,41,42], whereas others did not observe significant improvements [14,43,44].

The same applies to fatigue as there is some evidence for an improvement of fatigue by endurance training [30,35,45], whereas other studies missed the level of statistical significance [14,28,35] or did not reveal any differences at all [27,46,47].

Contradictory data have been reported on various items of health related quality of life like vitality [14,48], social functioning [14,44,48], mood [14,42,44], energy [14,42], anger [14,41], sexual function [14], bladder and bowel function [41], and depression [14,41].

One group analyzed the effect of a 6 months outpatient aerobic training program in MS patients with mild to moderate disability (EDSS 1-6) and observed a trend for larger benefits in more severely disabled than in less affected patients, but the study is limited by the small sample size of 19 patients of which only 11 patients completed the study [42]. Therefore, these results have to be handled with care and further studies are required.

### Resistance training

Resistance training is known to enhance muscle strength in healthy people. In MS patients there is also evidence for improving muscle strength [35,40]. Furthermore, beneficial effects on walking speed, stepping endurance, stair climbing, timed up and go test, self-reported disability, and self-reported fatigue have been described in MS patients as well as significant improvements in gait disturbances, measured by Dynamic Gait Index [35,49].

There are different forms of resistance training. One form, for example, constitutes progressive resistance exercise (PRE), which according to Taylor et al. comprises the following three principles: "*1. perform a small number of repetitions with relatively high loads until muscle fatigue is reached, 2. allow sufficient rest between exercise for recovery, and 3. increase the load as the ability to generate muscle force development*" [40].

Cakit et al. examined the effect of PRE by means of cycling progressive resistance training and lower-limb strengthening, both combined with balance exercise in a prospective randomized controlled trial of 45 MS patients [35]. After 8 weeks, patients in the two training groups performed better with respect to 10 m walking test, duration of exercise, and timed up and go test than patients in the control group who received no intervention. Moreover, the training groups showed evidence for superior effects on balance, fatigue, depression, and fear of falling.

Taylor et al. investigated the effect of a 10 week PRE program on maximal muscle force, muscle endurance, functional activity, and overall psychological function in MS patients [40]. The authors reported significant improvements of arm strength, leg endurance, and fast walking speed, and a trend towards improvement in the 2-min walk-test and day-to-day life function.

Besides PRE, other training forms like strategies to promote proper gait mechanics, focusing on weight bearing, weight shifting, and body positioning, or weightlifting are used [49]. For example, Pilutti et al. examined the effect of resistance exercise in six severely disabled patients (EDSS 5-8) with progressive MS (five patients with primary progressive, one patient with secondary progressive disease course) by means of a 12 week course of body-weight supported treadmill training performed three times weekly for 30 min [32]. The patients improved in terms of training intensity treadmill walking speed and required body weight support as well as in physical and mental subscales of a quality of life questionnaire. Fatigue was not reduced.

### Combined endurance and resistance training

Only few authors examined the effect of combined resistance and endurance training in MS. Small improvements both in muscle strength and gait velocity have been described [14,34,50]. Interestingly, in a comparatively large study on 95 MS patients, Surakka et al. observed significant training effects after six months of combined resistance and endurance training only in women, but not in men, which might be explained by a 25% higher exercise activity in women [50]. Furthermore, Romberg et al. reported significant improvements in walking speed and upper extremity endurance following six months combined exercise training, whereas lower extremity strength, VO_2_-max, static balance, and manual dexterity did not improve [34].

In 2005, the Cochrane Collaboration published a first systematical review on the effects of exercise on ADL and health-related quality of life (HRQoL) and the effects of physical therapy on various symptoms in MS patients [33]. Only controlled, randomized clinical studies on adult MS patients not experiencing an exacerbation at the time were included. Six studies, of which four have so far only been published as an abstract, analyzed the effects of physical therapy (rehabilitation, physiotherapy, exercise, functional training, independent home-based training, aquatic exercise) on several disease-related variables compared to a control group that had not received any physical therapy [36,39,41,51-53]. Three other studies compared the results of two different physical therapy programs. In summary, muscle strength, movement (changing and maintaining posture, walking, moving around, timed transfer, walking cadence), and exercise tolerance tests (modified graded exercise test, VO_2_-max, and physiological cost index) all showed substantial improvement. Mood parameters (fear, depression) showed only moderate improvement and EDSS, fatigue, cognitive parameters and ADL remained unchanged [18,37,48].

Asano et al. assessed the methodological quality of selected randomized controlled trials (RCT) of exercise interventions in MS carried out from 1950 to 2007 [29]. They found evidence for positive effects of exercise on physical and psychosocial functioning and quality of life, but highlighted a great need for high quality RCTs in this field.

### Exercise in MS patients -- the impact of body temperature on disability

In 1890 the German ophthalmologist Wilhelm Uhthoff (1853-1927) first described visual impairment and paresis occurring after physical activity. Because the patients' body temperature was not recorded, Uhthoff assumed that the described symptoms were caused by the physical activity itself and not by the resulting increased body temperature. Consequently, MS patients were advised not to engage in exercise [14-16,19,46,54,55]. In fact, 60-80% of MS patients experience a reversible (re)occurrence or aggravation of neurological symptoms in situations with increased body temperature, for example during vigorous physical activity, fever, or a hot bath [14-16,46,54,55]. As a reference to the first description, the eponym "Uhthoff's phenomenon" has been coined. The underlying cause is thought to be a temperature dysregulation due to dysautonomia with subsequent temperature-dependent impairment of the conduction velocity of partially demyelinated axons [15,16,54,56]. Not until about 1937, numerous systematic investigations revealed the correlation between increased body temperature and aggravation of disability.

Another argument for MS patients to avoid exercise was the assumption that a "waste" of energy might aggravate fatigue and reduce ADLs [14] which however has never been confirmed. Furthermore, a detrimental effect of physical activity itself on CNS structures or an activity-mediated increase of the relapse rate has never been demonstrated [15,57].

### Exercise in MS patients -- effects on the immune system

It is well known that exercise may influence susceptibility to common infectious diseases like upper respiratory tract infections in different directions [58]. Whereas vigorous physical activity such as competitive sport can lead to an increased susceptibility to infections, moderate exercise may contribute to their prevention [15,19,57-59].

On the immune cell level, physical strain in healthy subjects has been demonstrated to initially increase the peripheral lymphocyte count which subsequently falls to below the initial level after cessation of the physical activity [19,60,61]. The resulting lymphocyte reduction was short-lasting with a maximum duration of 3-24 h [19,58,60] and was shown to be more prominent in Th1 cells than in Th2 cells [61-63]. As Th1 cells primarily secrete pro-inflammatory cytokines like IFN-γ, IL-2, and TNF-α whereas Th2 rather secrete anti-inflammatory cytokines such as IL-4, IL-5 and IL-10, exercise can promote a shift from a Th1-mediated pro-inflammatory to a rather anti-inflammatory Th2-mediated cytokine milieu [58,60] which is of particular interest because an imbalance of Th1- and Th2-cells is considered relevant in MS pathogenesis [62].

Since established immunomodulatory drugs such as IFN-β or glatiramer acetate exert similar effects on the immune system, drug treatment and physical activity may complement each other in terms of modulating the immune system. The only short lasting effects of exercise on the immune cell level argue for regular and frequent training intervals.

The effect of exercise on cytokine production and response is less clear and often contradictory [44,60,62,64], which can in part be explained by different populations studied, different training protocols and/or different readout parameters and paradigms. For example, Heesen et al. found similar resting serum concentrations of IFN- γ, TNF- α and IL-10 in trained and untrained MS patients [62], whereas White et al. reported reduced resting plasma concentrations of IL-4, IL-10, C-reactive protein (CRP) and IFN- γ and a tendency for decreased TNF- α in MS patients upon eight weeks of PRE. Muscle contractions are thought to stimulate secretion of IL-6 [44,65]. Likewise, contradictory data have been published on the effect of exercise on immunoregulatory IL-6 in MS patients [44,64].

Given the neurodegenerative component of MS, the effect of physical activity, particularly of exercise on nerve growth factors is of particular importance. In rodents, exercise has been shown to stimulate the release of brain-derived neurotrophic factor (BDNF) [66], insulin-like growth factor 1 (IGF-1) [67-69] and vascular endothelial growth factor (VEGF) [70], all of which support cell proliferation, synaptic plasticity, neuroprotection, and neurogenesis in both physiological and neuroinflammatory conditions [67,71-74]. Also in humans exercise seems to modify the secretion of neuroactive proteins [14,67]. In both healthy participants and MS patients 30 min of moderate ergometry-based exercise increased the concentrations of BDNF and nerve growth factor (NGF) [59,75]. Increased hippocampal BDNF concentrations have been measured upon moderate exercise [67]. Since the hippocampus is crucially involved in learning and memory tasks and modulation of mood, these findings might connect exercise with slowing of cognitive impairment and stabilization of affect in MS patients [67]. An increased secretion of IGF-1 has so far been demonstrated in healthy people after exercise [76-78]. IGF-1 as an important factor in development supports cell survival, brain growth and CNS myelination. During later phases of life IGF-1 might play a role in neuroprotection and synaptic and cognitive plasticity [67]. Furthermore, exercise increased the activity of antioxidant enzymes, which might support the role of exercise in neuroprotection [67].

### Exercise in MS patients -- effects on morphology and imaging findings

Repetitive activation of the motor programs strengthens the cortical engrams and causes neuroplastic and adaptive processes like improved motor unit activation and synchronization of firing rates. In contrast periods of inactivity are associated with opposite effects [35,49,79].

Although data on the effect of physical activity on brain structural parameters are sparse, some evidence indicates that physiotherapy and regular fitness training counteract the structural degeneration of brain tissue in patients with relapsing-remitting MS and possibly have a neuroprotective impact. Both grey and white matter atrophy occurs already in early stages of relapsing-remitting MS [80]. However, patients with a higher level of aerobic fitness were shown to have a comparatively larger local volume of grey matter in the right post-central gyrus and midline cortical structures including the frontal medial and the anterior cinguli gyrus and the precuneus somatosensory cortex than unfit patients. Furthermore higher fitness levels were associated with greater recruitment of cortical regions whereas lower fitness levels were associated with enhanced anterior cingulated cortex activity [81]. These data should however be treated with caution as they based on a small sample of 24 female MS patients with a wide range in disability (EDSS 0-6) and disease duration (1-18 years).

MS patients have been shown to have more brain areas, often bilaterally, activated when performing motor and cognitive tasks compared to healthy controls, possibly as an expression of neuroplasticity [82-92]. The degree of ipsilateral activation appears to correlate with the disease course and severity [85,88,93] and is considered to reflect cortical adaptive reorganization processes [82,85,86]. For example, in MS patients with primary progressive disease course movement-associated cortical activation involved "nonmotor" areas like the insula and several multimodal cortical regions in the temporal, parietal, and occipital lobes in addition to the "classic" areas of motor planning and execution regions (including the supplementary motor area and the cingulate motor area) [93]. Morgen et al. reported that thumb movements of untrained MS patients elicited a more prominent activation of the contralateral dorsal premotor cortex in fMRI than in healthy controls [85] which in contrast to healthy controls was not attenuated upon repetitive thumb movements.

In MS patients the corpus callosum is typically affected. Besides callosal lesions detected by standard MRI sequences, diffusion tensor imaging sequences show ultrastructural damage, reflected by a reduced fractional anisotropy and increased mean diffusivity [79,94-98]. Interestingly, in a small study comprising 11 MS patients and healthy controls, Ibrahim et al. described a significant increase of fractional anisotropy and mean diffusivity in the corpus callosum after a two months physiotherapy program of 2 h per week, suggesting that physiotherapy may influence the brain microstructure in MS [79]. In summary, some data suggest, that effects of exercise in MS patients may be reflected by morphological changes in the CNS which may be detectable by advanced imaging techniques. However, existing data are not yet sufficient to unequivocally prove an impact of exercise on brain structure in MS.

### Personalized exercise in MS patients -- general and specific recommendations

At the start of the 1990s the German Federal Health Monitoring System's general recommendation of performing a specific health-related training program at least three times a week was replaced by a more global perspective, namely the integration of everyday physical activities. In the situation of MS patients with an often reduced everyday activity, regular exercise is particularly important. Apart from improving muscle strength, exercise is intended to improve endurance, muscle tone and posture stability, the degree of flexibility, endurance and and should involve both the agonists and antagonists [15,35]. A physical training program needs to be tailored to the individual needs and symptoms of a patient. Factors to be considered include the course and stage of disease, the degree of disability, age, concomitant diseases and sequelae. Importantly, it has to be ensured that the patient is not overstrained [14-16].

Compared to healthy people MS patients have a reduced aerobic capacity [14,26,38], decreased muscle strength, retarded rate of muscle tension development, reduced muscle endurance and impaired balance [14,15,36,99-101]. A relationship between gait speed and strength parameters has been postulated [102]. Petajan and White illustrated the level of muscular fitness and physical activity of MS patients in two "pyramids": passive range of motion (ROM) forms the basis of the muscular fitness pyramid and can minimize the risk of contractures when practiced regularly [16]. The next step in the pyramid comprises active flexibility and resistance exercise against or without gravity to maintain muscle integrity, for example to enable the patient carrying out essential daily functions. A well-rounded program of muscle strengthening exercise represents the top of the muscular fitness pyramid [16]. ADLs form the basis of the physical activity pyramid, followed by built-in inefficiencies, active recreation, and structured aerobic training programs. Again, design, frequency, and intensity of training programs have to be tailored to the individual patient. Weight-supported exercises like ergometry and water exercise are particularly recommended for patients with motor deficit or balance disturbances [16].

No specific recommendations for exercise treatment exist that are universally valid. However, general therapeutic recommendations can be defined. Since exercise programs have not sufficiently been investigated in more severely disabled patients, these recommendations are restricted to MS patients with a maximum EDSS score of 7 [14,15,34,38]. Any new exercise program should be initialized by a physiotherapist or exercise physiologist familiar with the disease [14]. A brief history including impairments in particular within daily activities should be elicited [16]. Regardless of the type of exercise, training programs should be uncomplicated and comprehensible to the patients. If necessary, it might be advisable to explain training programs in an illustrated or written form [15]. Patients should be supervised until they can perform the program adequately and independently [14-16,26]. Exercise programs should specifically target weaker muscles, and should preferably encompass multisegmental complex movements [15,35]. The intensity should be increased only slowly, and not to the point of pain [15]. Special care should be paid to peripheral nerves; particularly overstretching should be avoided [15]. Training sessions are recommended to start at a low level, include a light warm-up, progress according to the patients' clinical state and specific problems, and finally reach light to moderate intensity [14-16,26]. 10-15 min of daily stretching to maintain and improve flexibility of muscles and tendons [15] and recovery time between training sessions of 24-48 h are recommended [15]. Immobilized patients or those with severe clinical symptoms should be individually assisted. Some authors advise that cardiopulmonary function and VO_2_-max should be assessed prior to treatment start since MS patients may have reduced heart rate responses in graded exercise testing, possibly as an expression of cardiovascular dysautonomia [15,16], although this probably can hardly be implemented in the daily routine. Regarding endurance training and according to the American College of Sports Medicine, White and Dressendorfer recommend using the actual heart rate response to graded exercise testing for finding the ideal target heart range for training [15]. No symptoms should appear and "moderate intensities" ought to be strived, for example by means of the Borg scale of perceived exertion, which ranges from 6 to 20 (6 means "no exertion at all", 20 means "maximal exertion"). For moderate intensities ranges from 11 to 14 are aspired [15,103]. Depending on the symptoms and the training program, exercises should be performed at home, individually, with a training partner, or with a training group, and may include training equipment such as elastic bands, additional weights and pulley systems. Due to its social support a training group seems to be favorable in terms compliance and motivation [16,28]. To achieve similar effects in home-based training programs, patients should be closely supervised, for example by visits or telephone calls [16,28]. Most importantly, the training sessions have to be performed regularly [14-16,26].

Some special recommendations regarding exercise training for MS patients have been published. However, it has to be emphasized that these recommendations mostly represent personal experiences made by the authors and are not always supported by high standard clinical trials. Dalgas et al., for example, recommended endurance training of approximately 10-40 min duration, with an initial training intensity of 50-70% of VO2-max corresponding to 60-80% of maximum heart rate [14]. According to Dalgas et al., resistance training is recommended to initially comprise 8-15 repetitions which can then be increased over several months. The training should start with 1-3 sets, later 3-4 sets with a 2-4 min break between sets and should be performed two or three times per week. For heat-sensitive patients and those who regularly develop Uhthoff's phenomenon exercise training in the morning or in water at temperatures of 27-28°C could be preferable since body temperature is physiologically lower early in the day and heat generated by physical activity is quickly dissipated in water [15,16]. Alternatively, cooling before exercise and/or during physical activity for example by cold packs may help to prevent Uhthoff's phenomenon [15,16,55]. Also, resistance instead of endurance training could be preferable for heat-sensitive patients [14].

## Physical therapy approaches to prevent or alleviate individual target symptoms and signs in MS

### Fatigue

Fatigue, defined as an extreme physical and mental tiredness inadequate to the preceding demand, is a frequent, often very debilitating symptom in MS, which is generally difficult to treat [8-10,15,35,104-106]. Approximately 75-90% of all MS patients experience fatigue during disease progression [8,10,16] and some MS patients end up in a vicious circle: out of a wish to reduce fatigue they decrease physical activity which over time reduces endurance, muscle strength, and quality of life and may enhance fatigue, which then thus in turn further limits physical activity and social life [9,42,49]. Apart from cooling, moderate exercise, particularly aerobic training, seems to have a positive effect on fatigue [30,35,45]. Because fatigue often increases over the day, training sessions should be performed in the morning and must not overexert the patient [104]. Special supports like participation in a training group or attending psychological support to increase motivation for continuation of training over time could be advantageous in patients suffering from fatigue [16]. Energy saving strategies are also applied, in which the patient learns to prior-itize and to perform everyday tasks with a minimum of exertion [4,16,27]. Although a beneficial effect of moderate exercise on fatigue has been described by some authors [14,28,35,41], effects are usually insufficient to achieve significant improvements in current fatigue scales [17,35,45,47,50]. Other studies completely failed to detect any improvements [33]. One explanation for contradicting results can be found in the use of different fatigue scales, which focus on physical symptoms, or in attendant sleep disturbances such as insomnia, sleep related breathing disorders, restless legs syndrome, periodic limb movement disorder [104-106]. In conclusion, there is some however not unequivocal evidence for low to moderate beneficial effects of moderate exercise on fatigue.

### Spasticity

With a lifetime prevalence of about 90% spasticity is frequent in MS and has a potential to significantly reduce quality of life [104]. It leads to limitations in the range and normal pursuit of movements, results in malpositioning of the joints, and is often accompanied by pain [24]. Controlled studies on exercise and physiotherapy for MS-related spasticity are rare; however some evidence for improvements has been reported [104].

Physical therapy measures include active and passive exercise (e.g. targeted positioning of the patient, passive exercise using motorized cycles, active treadmill exercise) which can be assisted by a training partner or training equipment such as elastic bands. Physiotherapeutic techniques according to Bobath or Vojta and proprioceptive neuromuscular facilitation (PNF) are among the treatments applied. None of these measures has been proven to be superior [104,107]. It is most important to carry them out regularly and with a sufficient intensity [4,104]. Light stretching of the affected muscle groups with duration of approximately 20-60 s should be performed prior to and after exercise [15].

### Pareses

Pareses lead to various physical disabilities, such as difficulty in walking and fine-motor dysfunction. A relationship between gait speed and muscle strength in MS patients has been shown [14]. As no drug treatment for pareses exists and antispastic drugs such as baclofen may also lead to a worsening of existing pareses, physical and occupational therapy techniques are the sole treatment option. Because of reduced impact of gravity aquatic training allows patients with even severe pareses of the lower extremities to perform standing and moving exercises [15,16]. A standing frame can help patients who are unable to stand, to train torso, limb, and respiratory muscles and protects against cardiovascular dysregulation. For immobilized patients, passive range of motion exercises proximal to the paralyzed region is recommended [15,16]. Various studies have shown a significant improvement of muscle strength due to exercise [33,35,40,101]. Furthermore some authors reported beneficial effects in walking speed, stepping endurance, stair climbing, and timed up and go test [35,40,49]. In summary, evidence suggests that exercise is beneficial in the treatment of MS-related pareses, however again, only few, partially inconsistent data are available. Moreover, effects of exercise have been studied almost exclusively in MS patients with mild or moderate impairment.

### Coordination and balance dysfunction

Abnormalities in balance control are frequent symptoms in MS patients, which restrict patients in their daily living activities and increase risk of falls [5]. Balance skills like standing and walking, as well as the patients' perception of their own balance are important to assess [5]. The sitting position of cycling training is advantageous for unsteady patients [15,16]. Only a few studies investigated the influence of exercise programs on balance and coordination in MS and very few have chosen these variables as primary outcome parameter. Catteneo et al., for example, investigated the effect of balance training in 44 MS patients in a randomized controlled trial [5]. Two treatment groups received particular balance rehabilitation for three weeks, a third (control) group participated an unspecific training program. In both treatment groups a reduction of the number of falls and an improvement in clinical tests of static balance (Berg Balance Scale) and dynamic balance (Dynamic Gait Index) could be detected. However, in self-assessment scales patients did not report significant improvements [5]. Another controlled study did not support a beneficial effect of exercise training on static balance [34].

### Cognitive and mood disturbances

Depending on the disease course and stage 45-70% of MS patients are affected by cognitive impairments like reduced information processing speed, attentional deficits and episodic memory deficits [12,13,24,104,108] and 60-70% experience mood disturbances [13,109,110]. Some evidence for a positive correlation between aerobic exercise and cognition and brain function in healthy people has been described [81]. In MS patients, beneficial effects of regular physical activity and exercise on mood [18,32,35,48] and quality of life [14,15,28,34] have been repeatedly reported. Valid data on the effect on cognitive function are hardly available.

## Conclusion and outlook

Several lines of evidence suggest that MS patients benefit from regular physical activity and exercise with respect to clinical, imaging and physiological parameters. However, the quality of so far realized clinical trials on exercise training in MS do not always satisfy the requirements of a high standard study. Moreover, because of different treatment paradigms and endpoints, data are often hardly comparable. Thus, many questions remain still unanswered. In consequence, there is a great need for standardized high quality and well described studies that address both short and long term effects of exercise on clinical and paraclinical parameters in MS patients with different disease courses and different grades of disability.

## Conflicts of interests

The authors declare that they have no competing interests.
